# New green strategies for labelling of glycoprotein *N*-glycans

**DOI:** 10.1039/d6ra05264a

**Published:** 2026-07-20

**Authors:** Emily Harlin, Fergal Byrne, Robert B. P. Elmes, Róisín O'Flaherty

**Affiliations:** a Dept. Chemistry, Maynooth University Maynooth Co. Kildare W23F2H6 Ireland roisin.oflaherty@mu.ie; b Kathleen Londsdale Institute for Human Health, Maynooth University Maynooth Co. Kildare W23F2H6 Ireland; c SSPC – The Research Ireland Centre for Pharmaceuticals, University of Limerick Castletroy Co. Limerick V94 T9PX Ireland; d CÚRAM, SFI Research Centre for Medical Devices, Biomedical Sciences Building, University of Galway H91 W2TY Ireland

## Abstract

2,2,5,5-Tetramethyloxolane (TMO) and Cyrene are recognised as sustainable, bio-based solvents that match the analytical performance of acetonitrile (MeCN) for fluorescent labelling of *N*-glycans. Eight solvents from dipolar aprotic, non-polar, and polar protic classes were systematically evaluated for compatibility, labelling efficiency, and reproducibility in the fluorescent labelling of *N*-glycans using 6-aminoquinolyl-*N*-hydroxysuccinimidyl carbamate (AQC) from two glycoprotein standards, namely Immunoglobulin G and Fetuin. Hydrophilic interaction ultra-performance liquid chromatography (HILIC-UPLC) was used for visualisation and quantification of fluorescent *N*-glycans. Absolute quantification was achieved using a spiked internal standard, enabling picomolar-level quantification and facilitating direct comparison across solvents. TMO and Cyrene maintained glycan profiles, resolution, and peak intensity comparable to MeCN, while offering enhanced safety and environmental benefits. The workflow demonstrates that greener solvent systems can be incorporated into glycoanalytical pipelines without compromising performance, providing a scalable, sustainable alternative for biopharmaceutical and glycoscience applications.

## Abstract 2

2,2,5,5-Tetramethyloxolane (TMO) and Cyrene are recognised as sustainable, bio-based solvents that match the analytical performance of acetonitrile (MeCN) for fluorescent labelling of *N*-glycans.

## Graphical abstract

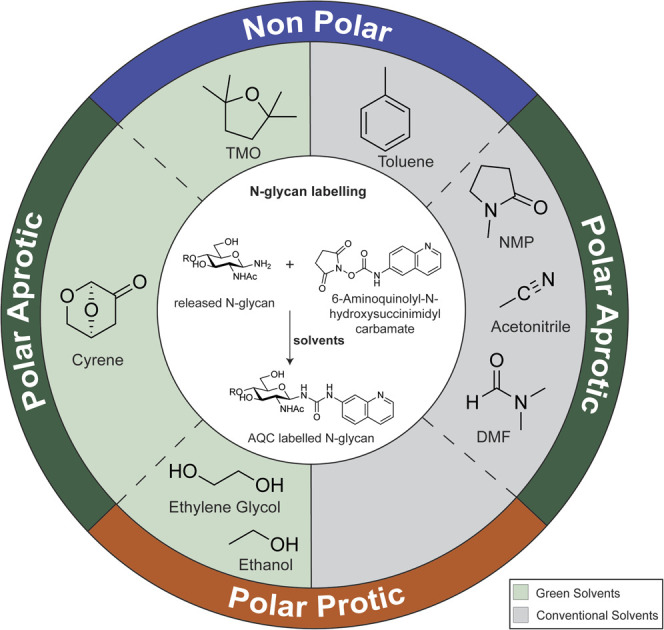

## 1. Introduction

Sugars, also known as glycans, are important complex molecules that can be covalently attached to proteins, lipids, or other biomolecules. *N*-glycans are an important class of glycans attached to asparagine residues within the consensus sequon Asn-X-Ser/Thr, where X can be any amino acid except proline, through an *N*-glycosidic bond. Their structures range from simple oligomannose forms to complex, branched species that may be fucosylated or sialylated.

Over 50% of human proteins are glycosylated, and over 80% of biopharmaceuticals contain *N*-glycosylation.^1^ Glycans are often altered in diseases involving immune dysregulation, including chronic inflammation, autoimmune disorders, and rare diseases.^1–3^ Glycosylation can notably affect the safety and efficacy of therapeutic proteins such as monoclonal antibodies, recombinant enzymes and glycoprotein hormones.^1^ As a result, robust tools for characterising *N*-glycans are crucial for both the Glycoscience and Biopharmaceutical communities.

**Fig. 1:**
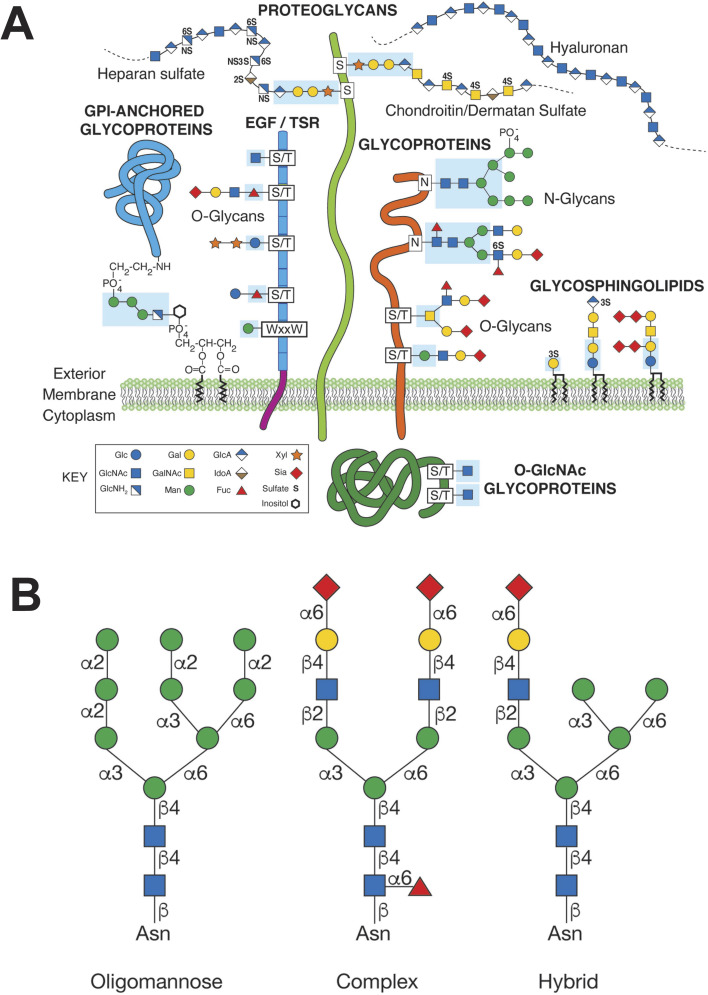
Major classes of animal glycans and structural types of *N*-glycans. (A) Schematic representation of the major classes of glycans found in animal cells, including glycosaminoglycans, glycoproteins, glycolipids, glycosylphosphatidylinositol (GPI)-anchored glycans, proteoglycans, and *O*-GlcNAc-modified proteins. (B) Representative structures of the three principal *N*-glycan classes: oligomannose, complex, and hybrid *N*-glycans. All *N*-glycans share the conserved Man_3_GlcNAc_2_ core attached to asparagine residues of glycoproteins, while differing in the composition and branching of terminal sugar residues. Taken from Varki *et al.*^7^

Technologies for *N*-glycan characterisation are well established in the literature and encompass workflows based on intact glycoproteins, glycopeptides, and released glycans.^4–6^ Among these, released *N*-glycan workflows are the most commonly employed. Standard released *N*-glycan workflows typically involve protein denaturation to disrupt higher-order protein structure, reduction and alkylation of disulfide bonds to improve enzyme accessibility, and enzymatic release of *N*-glycans using an aminidase such as PNGase F, which cleaves between the innermost GlcNAc residue and the asparagine residue of the glycoprotein backbone (Fig. 1).

Following enzymatic release, *N*-glycans are typically fluorescently labelled to enable chromatographic detection, as free glycans are not inherently fluorogenic. Excess label is then removed using solid-phase extraction (SPE), and the purified labelled *N*-glycans are analysed by hydrophilic interaction liquid chromatography-ultra-performance liquid chromatography with fluorescence detection (HILIC-UPLC-FLD).^1,8^ This workflow is frequently combined with complementary techniques, includingmass spectrometry, lectin arrays, and exoglycosidase sequencing to support structural and compositional characterisation of *N*-glycans.^9,10^

Many glycoanalytical procedures still rely on acetonitrile (MeCN), a polar aprotic solvent widely used in laboratories due to its favourable physicochemical properties, including polarity, water miscibility, low boiling point, and low UV cut-off.^11^ However, MeCN is classified as a problematic solvent in the CHEM21 solvent selection guide due to concerns about toxicity and sustainability, with toxicity linked to the metabolic release of cyanide.^12^ Since there is limited information on the long-term effects, including chronic toxicity and carcinogenicity, of prolonged use, the safety of routine laboratory applications is uncertain and may pose long-term health risks.^12^ In parallel, regulatory pressures, including REACH, are driving the need for safer and more sustainable alternatives in analytical chemistry.^13^ The development and adoption of green solvents have become a priority, with emphasis on reducing environmental and human health impacts. In 2023, Europe produced 68 million tonnes of environmentally hazardous chemicals and 167 million tonnes of hazardous chemicals to human health.^14^ Green solvents, defined by their reduced environmental impact, are assessed using frameworks such as the CHEM21 solvent selection guide, which evaluates safety, health, and environmental credentials.^15^

The shift towards sustainable analytical chemistry requires solvent substitution strategies that maintain analytical robustness without compromising environmental or human safety. However, solvent sustainability in fluorescent *N*-glycan labelling has not yet been thoroughly explored. This research addresses this gap by comparing conventional and bio-based solvent systems, using MeCN as a starting point for the Glycoscience and the Biopharmaceutical communities, and integrating sustainability assessment with a quantitative evaluation of labelling performance.

## 2. Results

As glycoanalytical workflows continue to grow in scale and complexity, it is timely to re-assess established methodologies through the lens of green chemistry principles. In this study, we focused on fluorescent labelling of enzymatically released *N*-glycans using 6-aminoquinoline-*N*-hydroxysuccinimidyl carbamate (AQC), a glycosylamine-reactive label used to enable chromatographic detection and quantification of released glycans.^16–18^ Accordingly, we examined eight solvents selected to represent a broad range of solvent properties and sustainability profiles, including polar aprotic, non-polar, and polar protic systems. The solvent panel comprised conventional solvents commonly employed in analytical and labelling workflows, including DMF, NMP, and MeCN; bio-derived or greener alternatives, including Cyrene, TMO, ethanol, and ethylene glycol; and toluene as a representative non-polar aromatic solvent. This selection enabled assessment of how solvent polarity, proticity, and sustainability profile influence AQC labelling efficiency, using the established MeCN-based protocol as the benchmark.

**Fig. 2:**
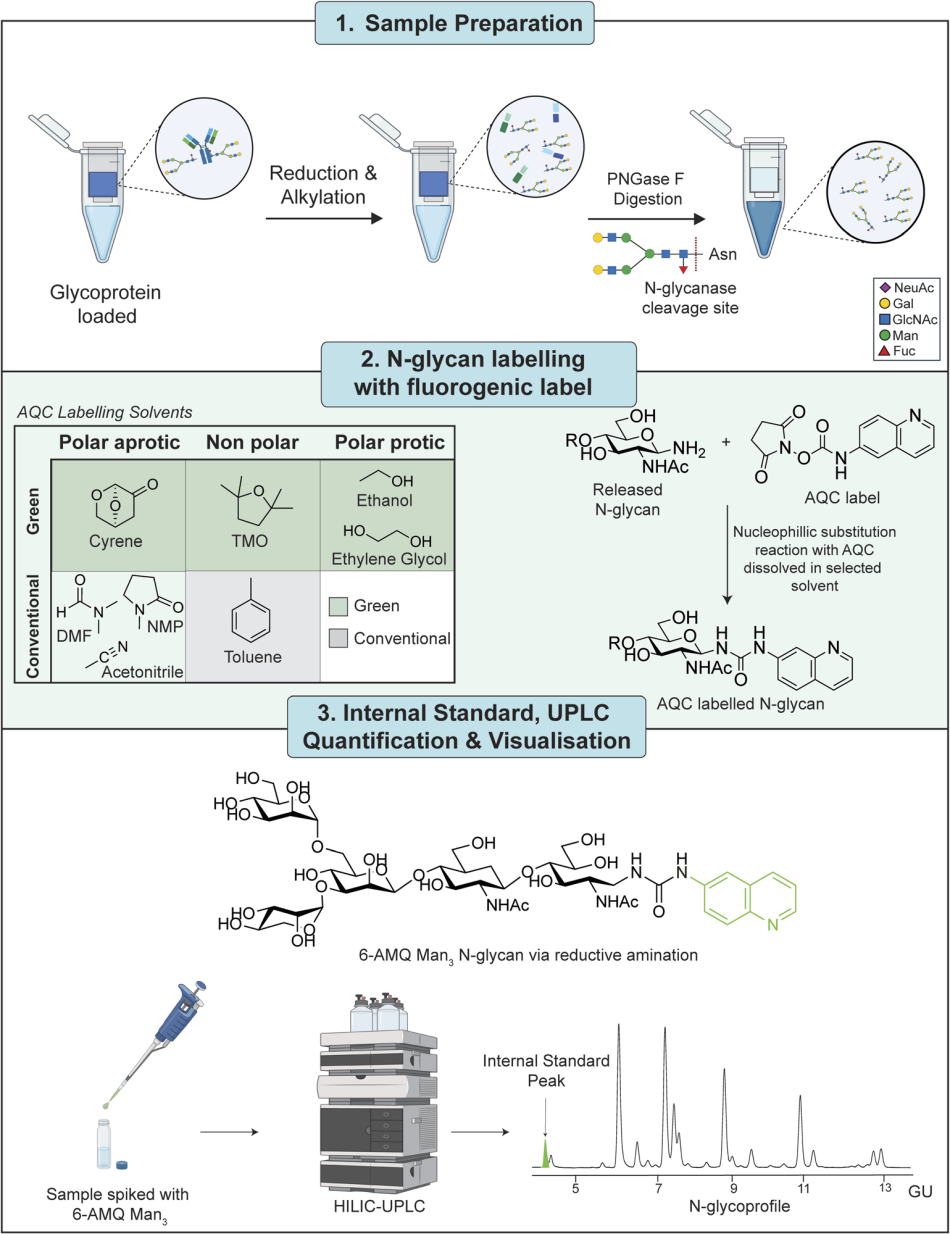
Experimental workflow of *N*-glycan release, AQC labelling, UPLC analysis with evaluation of organic solvents in the AQC labelling reaction and absolute quantification. (1) Following reduction, alkylation, and PNGase F digestion, *N*-glycans were released into an aqueous solution using a literature protocol.^19^ (2) The released *N*-glycans were labelled with AQC dissolved in selected organic solvents spanning different polarity classes (green and conventional: polar aprotic, non-polar, and polar protic). (3) Samples were spiked with fluorescently labelled Man_3_ internal standard (AMQ-Man_3_) of known concentration and analysed by HILIC-UPLC with fluorescence detection.

*N*-Glycans were released from human IgG and bovine Fetuin using previously established glycoanalytical workflows, with minor modifications (Fig. 2).^19^ Briefly, glycoprotein standards were denatured, reduced and alkylated, followed by enzymatic *N*-glycan release using PNGase.^19^ The released glycans were then fluorescently labelled with AQC and analysed by HILIC-UPLC with fluorescence detection, consistent with published AQC-based and released *N*-glycan analytical approaches.^16–19^ In the present study, alternative solvents were introduced specifically during the AQC labelling step, while glycan release, clean-up, chromatographic separation and detection conditions were otherwise maintained to allow direct comparison with the established MeCN-based workflow. Human IgG and bovine Fetuin were selected as well-characterised model glycoproteins representing relatively simple and more complex, highly sialylated *N*-glycan profiles, respectively.^5,18,19^

To support quantitative comparison between solvent systems, an AMQ-labelled Man3 internal standard was spiked into each sample prior to UPLC analysis. Man3 was selected as a structurally relevant *N*-glycan internal standard that elutes early in the HILIC-UPLC chromatogram. The concentration of the labelled internal standard was kept constant across all solvent conditions, enabling comparison of glycan concentrations and fluorescence response between solvent systems. This approach allowed solvent-dependent differences in derivatisation efficiency to be assessed using absolute quantification, in addition to the relative glycan profiling commonly used in released *N*-glycan workflows.

**Fig. 3:**
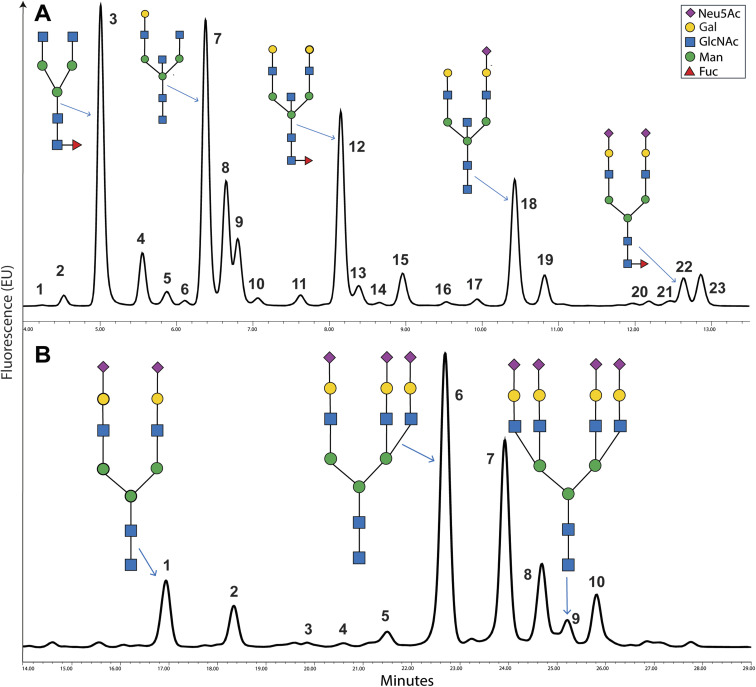
Representative HILIC-UPLC chromatograms of (A) IgG and (B) Fetuin following PNGase F release and AQC labelling. Fluorescence intensity (EU) is plotted against retention time (min). Glycan peaks were numbered according to elution order and integrated for quantification (IgG: 23 peaks; Fetuin: 10 peaks). Peak assignments were based on previously reported glycan characterisation studies.^18,19^ Glycan structures are depicted using Symbol Nomenclature for Glycans (SNFG),^21^ where symbols represent monosaccharides as follows: *N*-acetylglucosamine (GlcNAc), mannose (Man), galactose (Gal), fucose (Fuc), and *N*-acetylneuraminic acid (Neu5Ac), with sialylation indicated by Neu5Ac residues.

*N*-Glycans were released from IgG and Fetuin and analysed using a previously established glycoanalytical workflow^19^ with minor modifications. In short, IgG and Fetuin were denatured with dithiothreitol (DTT), alkylated with iodoacetamide (IAA), and *N*-glycans were enzymatically released using PNGase F as previously described.^19^ The released glycans were subsequently labelled with 6-aminoquinolyl-*N*-hydroxysuccinimidyl carbamate (AQC). Alternative solvents to the commonly used MeCN were introduced solely during the AQC fluorescent labelling step for human IgG and bovine Fetuin, respectively. Excess label was removed by solid-phase extraction (SPE), after which samples were dried and reconstituted in HILIC starting conditions (MeCN/water). The *N*-glycans were then separated and quantified by HILIC-UPLC. For IgG quantification, 23 glycan peaks (GPs) were integrated according to a literature protocol^19^ with assignments based on a previous literature reference (raw data in SI Tables S1).^10^ For Fetuin, 10 characteristic GPs were integrated; data aligned with literature ref. 5 and 18. Representative chromatograms are shown in Fig. 3. For both glycoprotein standards, relative abundances were expressed as percentages of peak areas (SI Tables S2 and S4). From this data, glycan traits, defined as quantitative summary measures of glycan profiles that capture specific structural features, such as galactosylation or sialylation, by grouping glycans with shared characteristics into a single interpretable value, were derived. These traits were generated from UPLC data (SI Tables S2 and S4) and calculated as the sum of individual GPs for each glycoprotein. They reflect key structural features, such as galactosylation and sialylation, and were calculated and adapted from a literature protocol.^20^ In contrast to the original method, which used relative percentage peak areas, this study calculated these traits from absolute peak areas obtained by chromatographic integration. For Fetuin, glycan traits were determined from the integrated peak areas of the 10 GPs and expressed as ratios reflecting relative sialylation, with the calculation methods detailed in the SI (Table S4).

For IgG, the efficiency of labelling in the alternative solvents and the method's reproducibility were assessed by comparing glycan traits that describe the relative abundance of agalactosylated (G0), monogalactosylated (G1), and digalactosylated (G2) glycans, as well as absolute peak areas, percentage areas, and glycan profiles obtained by HILIC-UPLC analysis (raw data and definitions of glycan peaks, traits, and derived traits are detailed in SI Tables S2 and S4). Alternative solvents replaced MeCN only during the AQC labelling step. Glycan release, chromatographic separation, and detection conditions remained otherwise unchanged. Enzymatically released *N*-glycans (PNGase F) were in aqueous solution, while AQC was dissolved in each test organic solvent (3 mg mL^−1^). Labelling, therefore, took place in a mixed aqueous–organic reaction environment. Each solvent condition was analysed in triplicate (*n* = 3), and reproducibility was evaluated by measuring the mean, standard deviation (SD), and coefficient of variation (CV).^22^ For IgG, CV values for the glycan traits ranged from 0.45–5.77%, while for Fetuin, they ranged from 0.06–3.62%. These values are well within the accepted limit of analytical precision (≤20% CV) as described in ICH guidelines,^22^ indicating excellent intra-day reproducibility across the tested solvent systems. This design facilitated direct assessment of solvent performance whilst maintaining comparability with existing glycoanalytical workflows. For IgG, reproducibility (*n* = 3) was assessed using glycan traits derived from absolute peak areas. The traits G0/G1, G0/G2, G0/S1, and G0/S2 were utilised to assess changes in galactosylation and sialylation across solvent systems. The G0/G1 traits compare agalactosylated structures with monogalactosylated species, while G0/G2 compares agalactosylated glycans with digalactosylated ones, reflecting galactose incorporation. The G0/S1 and G0/S2 traits compare agalactosylated glycans with mono- and disialylated species, respectively, giving an additional measure of glycan sialylation. Definitions and calculations of these traits are detailed in SI Table S2. For Fetuin, overall sialylation was assessed using the traits S2/S4 and S3/S4, which compare the relative abundance of di- and trisialylated glycans with tetra-sialylated species (S4). These traits provide an overview of glycan sialylation complexity, with higher values indicating a relative enrichment of less sialylated structures. Definitions and calculations of these traits are detailed in SI Table S4.

**Fig. 4:**
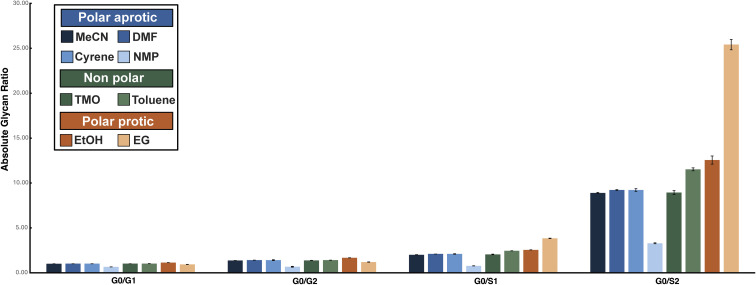
Absolute IgG *N*-glycan traits (G0/G1, G0/G2, G0/S1, and G0/S2) obtained following AQC labelling and HILIC-FLD analysis across the evaluated solvent systems. Solvents are grouped according to polarity class: polar aprotic (MeCN, Cyrene, DMF, NMP), polar protic (EtOH, EG), and non-polar (TMO, toluene). Data are presented as mean trait values (*n* = 3); error bars represent standard deviation. Coefficients of variation for all solvent conditions ranged from 0.06–3.60% (SI Table S2).

**Fig. 5:**
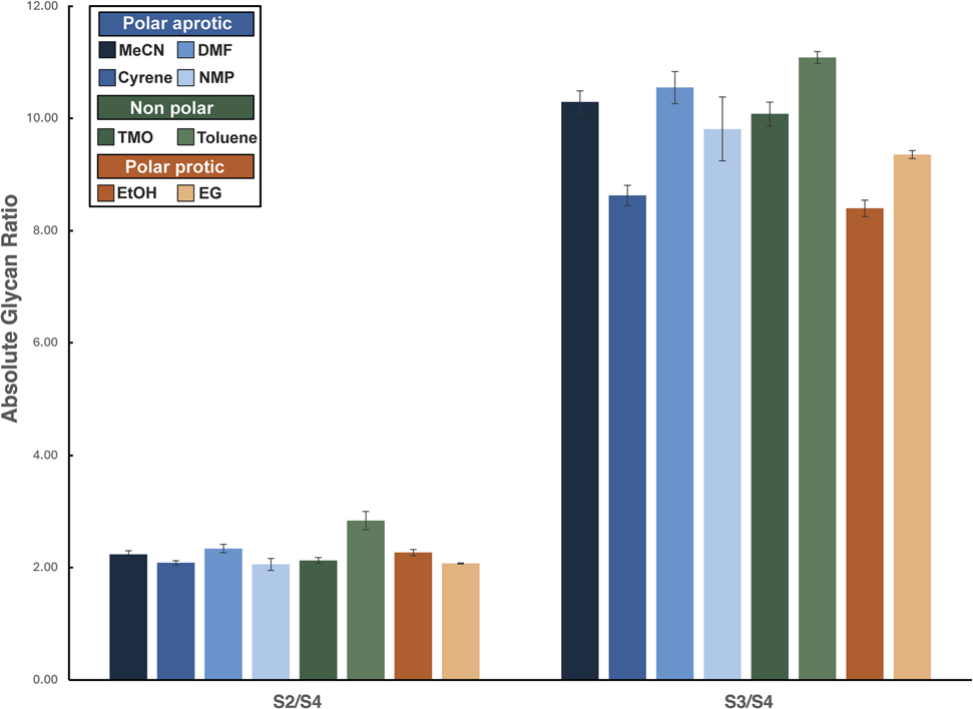
Absolute Fetuin *N*-glycan traits (S2/S4, S3/S4) obtained following AQC labelling and HILIC-FLD analysis across the evaluated solvent systems. Data are presented as mean trait values (*n* = 3); error bars represent standard deviation. Coefficients of variation for all solvent conditions ranged from 0.45–5.71% (SI Table S4).

Within the polar aprotic solvent group, Cyrene closely reproduced the glycan traits obtained with the MeCN reference, showing nearly identical behaviour across all IgG traits (Fig. 4). For example, the G0/G1 trait was essentially unchanged between the two solvents (1.00 in MeCN *vs.* 1.01 in Cyrene). In contrast, DMF and NMP produced systematic shifts in G0-based traits, with NMP showing the greatest deviation (G0/G1 = 0.66). These differences became more pronounced for traits involving sialylated glycans. The full dataset underlying these traits is provided in SI Table S2. For Fetuin, glycan traits remained highly consistent across the same solvent systems, with only minor variation observed for the S3/S4 metrics (Fig. 5). Overall, Fetuin sialylation was largely unaffected by solvent choice within this group. The full dataset underlying these traits is provided in SI Table S4.

Extension to the non-polar solvent group revealed a similar pattern. TMO closely matched the IgG glycan traits obtained with the MeCN reference, with only minimal differences observed across all measured glycan traits (Fig. 4). For example, the G0/G1 trait was nearly identical between the two solvents (1.01 in TMO *vs.* 1.00 in MeCN). In contrast, toluene produced modest increases in traits involving sialylated glycans, whereas traits comparing neutral glycans remained largely similar to those in MeCN. This trend was also observed for Fetuin, where the sialylation traits S2/S4 and S3/S4 remained closely aligned with MeCN in TMO but showed slightly higher values in toluene. Evaluation of the polar protic solvent group revealed solvent-dependent differences with respect to labelling in IgG glycan traits (Fig. 4). Compared with the MeCN reference, ethanol produced modest increases in glycan traits, for example, G0/G1 increased to 1.13 *vs.* 1.00 for MeCN. In contrast, EG showed larger changes in traits involving sialylated glycans, most notably for G0/S2, which increased to 25.42 in EG compared with 8.88 in MeCN. These differences became more pronounced for traits involving increasingly sialylated species. For Fetuin, the corresponding sialylation traits remained broadly consistent across the same solvent systems, with S2/S4 and S3/S4 values remaining close to those observed for MeCN. The full dataset underlying these traits is provided in SI Tables S2 and S4.

Across all solvent classes, glycan trait analysis reveals clear protein-dependent solvent effects with respect to labelling. IgG displays pronounced solvent sensitivity, particularly in G0-based traits containing sialylated glycans, with deviations increasing alongside glycan complexity in certain solvents, whereas Fetuin, a largely sialylated glycoprotein remains comparatively stable with only modest and inconsistent variation. These trends align with the individual glycan peak data reported as percentage peak areas (*n* = 3) in the SI (Tables S1 and S3). In addition to relative quantification of glycan distributions, absolute quantification was performed using an AMQ-labelled Man_3_ internal standard to determine if one solvent yields more fluorescent glycans. While relative glycan traits remained largely consistent across several solvent systems (SI Tables S2 and S4), absolute quantification revealed significant solvent-dependent differences in actual derivatisation efficiency.

Absolute concentrations of representative glycans from IgG (GP3, GP7, and GP18) and Fetuin (GP1, GP6, and GP9) were determined using AMQ-Man_3_ as an internal standard. The suitability of AMQ-Man_3_ for quantification was supported by external calibration experiments demonstrating a linear relationship between AMQ-Man_3_ amount and fluorescence response (*R*^2^ = 0.9972; Fig. S1 and Table S5). Mean concentrations are summarised in Table 1, with the full quantitative datasets available in SI Tables S2 and S4.

**Table 1:** Absolute concentrations of representative IgG and Fetuin *N*-glycans determined using the Man_3_-AMQ internal standard under different solvent conditions. Values represent calculated concentrations derived from internal standard normalisation (*n* = 3), where higher measured glycan concentrations correspond to more efficient labelling. Cells are colour-coded according to the order of magnitude of the calculated glycan concentrations relative to those obtained in the MeCN reference. Dark blue indicates concentrations consistent with the same order of magnitude as MeCN; intermediate blue indicates values approximately one order of magnitude lower (×10^−10^ mol L^−1^); and light blue indicates two orders of magnitude lower (×10^−11^ mol L^−1^). Full datasets and associated variability are provided in SI Tables S2 and S4

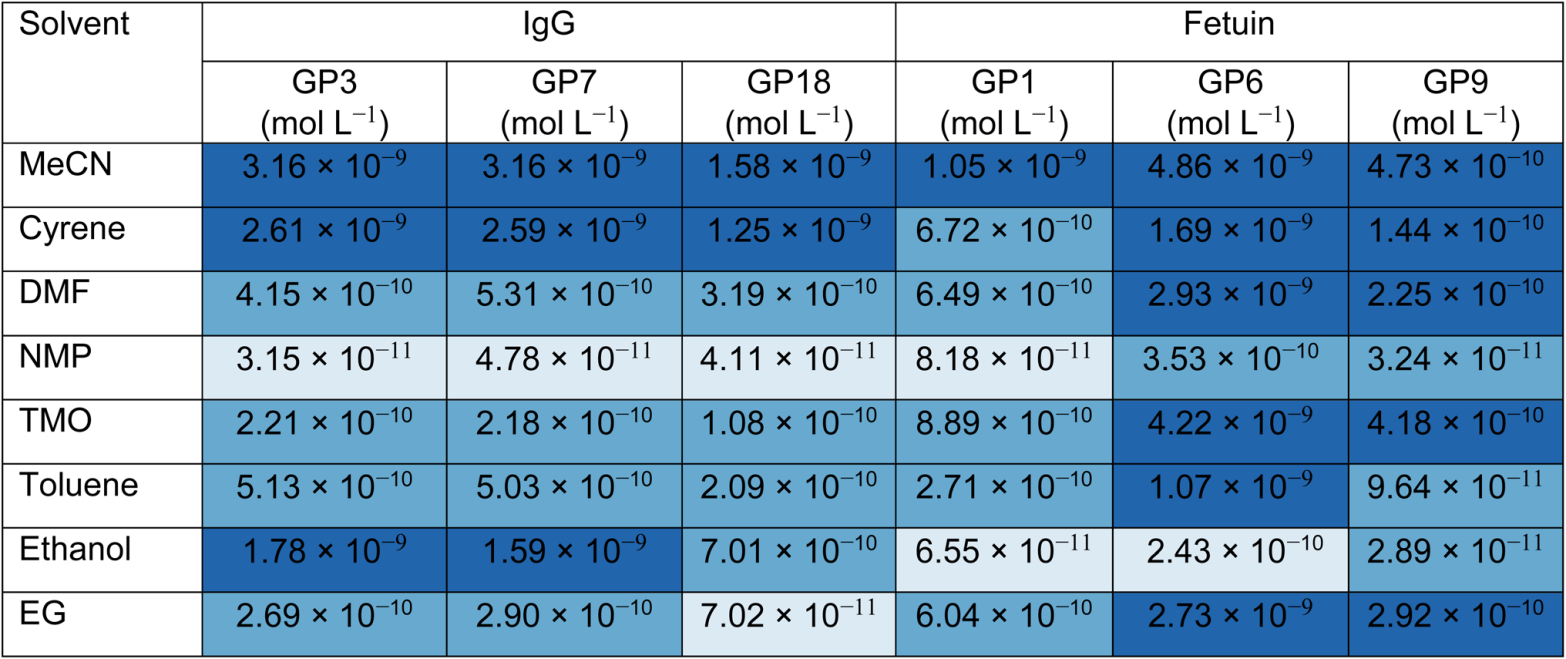

Absolute glycan concentrations determined by internal standard quantification (Table 1) followed the same solvent-dependent trends asobserved for the glycan trait analysis. Under the MeCN reference conditions, both IgG and Fetuin glycans were consistently quantified at concentrations on the order of 10^−9^ mol L^−1^ (*e.g.*, IgG GP3 = 3.16 × 10^−9^ mol L^−1^; Fetuin GP1 = 1.05 × 10^−9^ mol L^−1^). Cyrene produced concentrations within the same order of magnitude for both glycoproteins (*e.g.*, IgG GP3 = 2.61 × 10^−9^ mol L^−1^; Fetuin GP1 = 6.72 × 10^−10^ mol L^−1^), indicating comparable derivatisation performance to the MeCN reference. Several additional solvents, including EtOH and TMO, also produced concentrations within the ×10^−10^ mol L^−1^ range, whereas DMF, toluene, and ethylene glycol generally yielded concentrations approximately one order of magnitude lower. In contrast, NMP produced the lowest quantified concentrations across both glycoproteins, with values decreasing to the 10^−11^ mol L^−1^ range.

Overall, the absolute quantitation results closely mirrored the trends observed in the glycan trait analysis. Using the Man_3_-AMQ internal standard, glycan concentrations were consistently quantified across both glycoproteins over multiple solvent systems, demonstrating robust analytical response and enabling detection at low picomolar levels. While MeCN served as the reference condition, several alternative solvents, most notably the bio-derived solvent Cyrene and TMO, yielded glycan concentrations within comparable ranges across multiple glycans for both IgG and Fetuin. These results highlight the strong quantitative performance achievable in these alternative solvent systems. As the concentration of the internal standard remained constant across all experiments, the observed differences reflect solvent-dependent variation in derivatisation efficiency and analytical response rather than differences in analyte loading. Full quantitative datasets, including mean concentrations and associated variability, are provided in the SI Tables S1–S4.

## 3. Discussion

Several greener solvent systems can enable efficient AQC-based fluorescent labelling of *N*-glycans while delivering analytical performance comparable to that of the established MeCN workflow. Specifically, the bio-derived solvent Cyrene and the sustainable aromatic substitute TMO achieved glycan labelling efficiencies similar to the conventional MeCN benchmark for both IgG and Fetuin (SI Tables S2 and S4). These results demonstrate that greener solvent substitution in *N*-glycan derivatisation workflows can be achieved without compromising analytical performance, highlighting the potential for integrating more sustainable solvent systems into routine glycoanalytical methodologies. More broadly, the adoption of green solvents has gained increasing attention across industrial and separation technologies, where sustainable solvent substitution strategies can reduce environmental burdens while maintaining process performance.^23^ These broader developments further support the evaluation of environmentally preferable solvents within glycoanalytical workflows.

In the context of recent advancements in glycoanalysis, the shift from relative to absolute quantification is increasingly recognised as essential for developing reproducible biomarkers. A key methodological aspect of this study is the use of the AMQ-Man_3_ internal standard, which enables absolute quantification of glycans across all solvent systems. Although demonstrated here in a green-solvent framework, this approach has broader applicability in glycoanalysis, enabling more reliable and comparable quantitative measurements across different methods and platforms. While glycan traits appeared broadly consistent across several solvent systems, absolute quantification showed variation in measured glycan concentrations, potentially reflecting differences in derivatisation efficiency, sample handling, or analytical response. The use of an internal standard helps account for these contributions to be distinguished, providing a more robust basis for evaluating analytical performance. The workflow enabled quantification at low concentration levels using the AMQ-Man_3_ internal standard introduced at 8.7 × 10^−11^ mol/L per sample. Although direct comparison with mass spectrometry-based approaches is not straightforward, this sensitivity is comparable to or exceeds concentration ranges reported in recent glycomics studies.^24^ These results demonstrate that high-sensitivity glycan quantification can be achieved using UPLC-FLD, supporting the detection of low-abundance species without reliance on mass spectrometric methods. Collectively, these findings highlight the use of tailored internal standards as a robust and versatile strategy for quantitative glycoanalysis, with clear potential to improve standardisation across workflows. Beyond the current solvent comparison, such methods could contribute to improved standardisation in quantitative glycomics, where differences in sample preparation and analytical response often complicate comparisons between studies.

Among the non-polar solvents examined, TMO showed particularly strong agreement with the MeCN reference across both glycoproteins. Relative glycan traits for IgG and Fetuin remained highly consistent with those obtained under MeCN conditions, and absolute quantification confirmed that glycan concentrations in TMO were maintained within the same order of magnitude as the reference system. In contrast, the conventional aromatic solvent toluene produced modest deviations in several traits and slightly lower concentrations. The strong performance of TMO may reflect its distinctive chemical structure. Although immiscible with water and generally classified as non-polar, TMO contains an ether oxygen with notable hydrogen-bond accepting ability.^25^ Despite steric hindrance from its methyl substituents, this feature differentiates TMO from traditional hydrocarbon solvents such as toluene and may influence the local environment of the AQC labelling reaction.^26^ Together, these observations highlight TMO as a promising greener alternative to conventional aromatic solvents in *N*-glycan derivitisation workflows.

For IgG *N*-glycans, the observed differences between solvent systems may also reflect broader solvent-reaction interactions during the derivatisation step, potentially influencing neutral and sialylated glycans differently. Because the internal standard concentration was constant across all solvent systems and derivatisation solvents were removed prior to chromatographic analysis, these differences most likely reflect variation in AQC derivatisation efficiency rather than chromatographic effects. DMF and NMP are strongly coordinating polar aprotic solvents with relatively high Lewis basicity and donor numbers,^27^ thereby enabling strong solvation of charged reaction intermediates formed during the AQC derivatisation step. In contrast, MeCN and Cyrene are less strongly coordinating, potentially providing a less competitive solvation environment, thereby facilitating formation of labelled glycan products.^27^ This suggests that the strength of solvent coordination may influence the efficiency of AQC derivatisation.

Additional solvent-dependent behaviour was observed for the polar protic solvents ethanol and ethylene glycol. Relative glycan traits for both glycoproteins remained broadly comparable to the MeCN reference, although absolute quantification revealed lower glycan concentrations for several glycans in ethylene glycol (Table 1). These differences may reflect the physicochemical properties of the solvent, as ethylene glycol forms extensive hydrogen-bonding networks and exhibits substantially higher viscosity than ethanol or MeCN. Within the previously described mixed aqueous–organic reaction environment, such properties may influence the local reaction conditions or reduce effective mixing and diffusion during the rapid AQC derivatisation step, potentially limiting glycan labelling efficiency.

These solvent trends observed in the glycan traits were more pronounced for IgG than for Fetuin. Several IgG traits showed noticeable variation across solvent systems, whereas the Fetuin traits (S2/S4 and S3/S4) remained comparatively consistent. This difference likely reflects the glycan classes represented in each analysis. The IgG traits included both neutral and sialylated glycans, while the Fetuin traits were derived exclusively from sialylated species. As a result, the IgG traits may be more sensitive to solvent-dependent differences in derivatisation behaviour between neutral and charged glycans, whereas the uniformly sialylated Fetuin glycans exhibit more consistent responses across solvent conditions.

One caveat is that the impact of solvent substitution must be considered across the entire glycoanalytical workflow. Cyrene was explored as a sustainable mobile-phase alternative to traditional solvents in HILIC chromatography, where MeCN typically constitutes the largest proportion of solvent consumption. Although Cyrene has not yet been reported in HILIC chromatography, it has previously been used in reversed-phase HPLC (RP-HPLC), where Cyrene served as a co-eluent with ethanol in the mobile phase, another eco-friendly solvent.^28^ However, Cyrene's high viscosity and limited miscibility with water^28^ may restrict its application in HILIC-UPLC, which depends on water partitioning between the mobile and stationary phases.^29^ In our experiments, high backpressure was observed when introducing Cyrene into the workflow, even at very low flow rates (0.1 mL min^−1^). The column used was a BEH amide HPLC column with a 2.6 µm particle size. We hypothesise that the combination of small participle diameter and Cyrene's high viscosity leads to this increased backpressure.

Taken together, these results demonstrate that greener solvent systems, such as Cyrene and TMO, can enable efficient AQC-based derivatisation of *N*-glycans while maintaining strong analytical performance relative to the conventional MeCN workflow. Coupling these solvent systems with internal standard-enabled quantification provides a robust analytical framework for evaluating glycan labelling efficiency and detecting subtle solvent-dependent effects that may remain hidden in trait-based analyses. This combination of greener solvent substitution and improved quantitative methodology may contribute to the development of more sustainable and analytically robust glycoanalytical workflows.

## 4. Experimental section

### 4.1 Chemicals, reagents and equipment

Milli-Q water was used for all buffer preparations and washing steps, generated from the Millipore Milli-Q Integral 3 A10TOC system. HPLC Vials (0.3 mL PP Short Thread Micro-Vial) were purchased from Apex Scientific. Acetonitrile (MeCN, HPLC Grade), ethanol (EtOH), Cyrene, *N*-methyl-2-pyrrolidone (NMP), *N*,*N*-dimethylformamide (DMF), toluene and ethylene glycol were purchased from Honeywell. 2,2,5,5-Tetramethyloxolane (TMO) was sourced from Addible. dl-Dithiothreitol (DTT), IgG from human serum, Fetuin from bovine serum, iodoacetamide (IAA), sodium bicarbonate (SBC), and sodium dodecyl sulfate (SDS) were all purchased from Sigma-Aldrich. Formic acid, sodium cyanoborohydride (NaBH_3_CN), acetic acid (AcOH) and dimethyl sulfoxide (DMSO) were all purchased from Sigma-Aldrich. PNGase F was purchased from New England BioLabs, 6-aminoquinolyl-*N*-hydroxysuccinimidyl carbamate (AQC) from Biosynth Carbosynth, 6-aminoquinoline (AMQ) from Sigma Aldrich and Man-3a *N*-Glycan from Ludger Ltd, Nanosep with 10k Omega Centrifugal Devices and HyperSep Diol 100 mg Well Plates were purchased from Thermo Scientific. Glycan preparation and release were performed using a MultiTherm Shaker and Hettich Zentrifugen Mikro 200. Samples were measured using a Waters ACQUITY UPLC H-Class with Empower 3 Operating Software equipped with an ACQUITY UPLC Glycan BEH Amide Column (130 Å, 1.7 µm, 2.1 mm × 150 mm).

### 4.2 Human IgG and bovine Fetuin *N*-glycan release and labelling (AQC)

*N*-Glycans were released from human serum IgG (50 µG) and bovine serum Fetuin (50 µG) using a literature protocol (O'Flaherty *et al.* 2019)^10^ with the following modifications. *N*-glycans were released manually using a 10 kDa Nanosep centrifugal filter cartridge. Proteins were denatured using 0.5 M dithiothreitol (DTT) and alkylated with 0.18 M iodoacetamide (IAA). Enzymatic release was performed using PNGase F (0.416 µL; 25 mM sodium bicarbonate buffer; final concentration 208 U mL^−1^) at 37 °C for 1 h, followed by fluorescent labelling. A 40 µL volume of collected *N*-glycans in aqueous solution was reacted with 100 µL aminoquinolyl-*N*-hydroxysuccinimidyl carbamate (AQC) (3 mg mL^−1^) in MeCN. Excess label was cleaned using a HyperSep diol 96-well plate with 95% MeCN, and the *N*-glycans were collected in 20% MeCN. The final samples were dried down.

### 4.3 AQC labelling of *N*-glycans using alternative solvents

AQC labelling of released *N*-glycans was performed according to the standard procedure described above, with MeCN substituted by alternative solvents. 40 µL of each *N*-glycan pool was aliquoted into a centrifugal vial (1.5 mL). The AQC solution (3 mg mL^−1^) in the respective solvents were prepared and reacted with the released *N*-glycans from IgG or Fetuin. The solvents evaluated were MeCN (reference solvent), Cyrene, NMP, DMF, TMO, toluene, ethanol and ethylene glycol. All labelling reactions were conducted using the same reagent volumes, incubation time and temperature as described for the standard MeCN-based method.

### 4.4 Calibration of IgG and Fetuin *N*-glycans using AMQ labelled Man_3_*N*-glycan

Released Man_3_*N*-glycans of known concentration were used as an internal standard for absolute quantification. The Man_3_ glycans were first treated with 20 µL of 1% formic acid and incubated at room temperature for 40 min. The AMQ labelling reagent was prepared as follows: NaBH_3_CN (484 mg, 7 mmol) was dissolved in 5.4 mL of DMSO. AMQ (390 mg, 2 mmol) was dissolved in 2.3 mL of acetic acid. The two solutions were mixed gently over ice. Afterwards 5 µL of AMQ labelling reagent was added and incubated for 30 min at 65 °C, followed by shaking of the samples for 5 min (500 rpm) and incubating for another 1.5 h at 65 °C. Excess AMQ was cleaned using a 96-well HyperSep Diol plate with 95% MeCN, and the sample was collected in 20% MeCN. The final fluorescently labelled samples were dried down. Before analysis, IgG and Fetuin samples were spiked with 1 µL of AMQ-labelled Man_3_ internal standard at a known concentration prior.

### 4.5 Ultra performance liquid chromatography (UPLC) for *N*-glycan visualisation and quantification

UPLC separated AQC-derivatised *N*-glycans with fluorescence detection on a Waters Acquity UPLC H-Class instrument. The HILIC separations were performed using a Waters Bridged Ethylene Hybrid (BEH) glycan column, (130 Å, 1.7 µm, 2.1 mm × 150 mm) with 50 mM ammonium formate (pH 4.4) as solvent A and MeCN as solvent B. Waters Acquity UPLC H-Class was fitted with a 0.2 µm filter. The separation was performed using a linear gradient of 70–30% MeCN over 20 min at a flow rate of 0.561 mL min^−1^. An injection volume of 19 µL was prepared in 70% MeCN for each injection. Samples were maintained at 5 °C before injection, and the separation temperature was 40 °C. The fluorescence detection excitation/emission wavelengths were *λ*_ex_ = 245 nm and *λ*_em_ = 395 nm, respectively.

Chromatographic data were processed by integrating the peak areas of the 23 resolved IgG *N*-glycan species.^19^ For Fetuin, nine characteristic GPs were selected and integrated using a model informed by previously reported approaches but adapted for the present method.^5,18^ Absolute quantification was achieved by normalising individual glycan peak areas to the internal standard and calculating concentrations based on the known amount of AMQ-labelled Man_3_.

## 5. Conclusion

This study examined how solvent choice affects the efficiency of AQC-based *N*-glycan derivatisation, using two representative glycoproteins, IgG and Fetuin. Among the solvent systems tested, MeCN served as the analytical reference and consistently delivered the most reliable derivatisation performance. However, several alternative solvents yielded comparable analytical results. Notably, bio-derived solvents such as Cyrene and tetramethyloxolane (TMO) produced glycan traits and concentrations closely aligned with those obtained with MeCN, indicating their capability to support efficient AQC labelling while maintaining the relative glycan distributions. Conversely, more strongly coordinating solvents like DMF and NMP resulted in lower glycan concentrations, implying that solvent–solute interactions may affect the reaction microenvironment during derivatisation. Similarly, the behaviour of polar protic solvents, ethanol and ethylene glycol, suggests that hydrogen-bonding interactions and transport properties influence labelling efficiency. Specifically, the higher viscosity and extensive hydrogen-bonding network of ethylene glycol likely contribute to reduced derivatisation efficiency relative to MeCN. Despite these solvent-dependent variations in efficiency, the overall glycan distributions for both IgG and Fetuin remained largely consistent across the tested solvent systems. This indicates that solvent effects mainly impact derivatisation efficiency rather than the fundamental glycan profiles obtained from analysis. The inclusion of an internal standard enabled improved discrimination between solvent-dependent effects and analytical variability, strengthening confidence in cross-solvent comparisons. This approach also supported reliable quantification at low glycan concentrations, underscoring its utility as a robust strategy for quantitative glycoanalysis. Overall, these findings show that greener solvent alternatives, such as Cyrene and TMO, can achieve analytical performance comparable to that of MeCN in AQC-based glycan derivatisation. The possibility of using these environmentally friendly solvents without sacrificing analytical accuracy underscores the potential to incorporate more sustainable solvent strategies into established glycomics workflows.

## Author contributions

Conceptualisation: E. H., F. B., R. B. P. E., R. O. F.; methodology: E. H., R. B. P. E., R. O. F.; formal analysis: E. H., R. O. F.; investigation: E. H.; data curation: E. H., R. B. P. E., R. O. F.; writing – original draft: E. H., R. O. F.; writing – review and editing: E. H., F. B., R. B. P. E., R. O. F.; supervision: R. B. P. E., R. O. F.; project administration: R. O. F.; funding acquisition: E. H., R. B. P. E., R. O. F.

## Conflicts of interest

No conflict of interest.

## Supplementary Material

RA-OLF-D6RA05264A-s001

## Data Availability

All data is provided in the supplementary information (SI). Supplementary information is available. See DOI: https://doi.org/10.1039/d6ra05264a.
